# Butyrylcholinesterase and Acetylcholinesterase polymorphisms in Multiple Sclerosis patients: implication in peripheral inflammation

**DOI:** 10.1038/s41598-018-19701-7

**Published:** 2018-01-22

**Authors:** Marcella Reale, Erica Costantini, Marta Di Nicola, Chiara D’Angelo, Sara Franchi, Marco D’Aurora, Maria Di Bari, Viviana Orlando, Sabrina Galizia, Serena Ruggieri, Liborio Stuppia, Claudio Gasperini, Ada Maria Tata, Valentina Gatta

**Affiliations:** 1 Unit of Immunodiagnostic and Molecular Pathology, Department of Medical, Oral and Biotechnological Sciences, “G.d’Annunzio” University, Via Dei Vestini 31, 66100 Chieti, Italy; 2 Department of Medical, Oral and Biotechnological Science, “G.d’Annunzio” University, Via Dei Vestini 31, 66100 Chieti, Italy; 3 Department of Psychological, Health and Territorial Sciences, School of Medicine and Health Sciences, “G.d’Annunzio” University, Via Dei Vestini 31, 66100 Chieti, Italy; 4 Molecular Genetics, Unit, CeSI-Met, Via Luigi Polacchi 1, 66100 Chieti, Italy; 5 Department of Biology and Biotechnologies Charles Darwin, Research, Center of Neurobiology Daniel Bovet, Sapienza University of Rome, 00185 Rome, Italy; 6 Department of Neurosciences, San Camillo Forlanini Hospital, Rome, Italy

## Abstract

Multiple Sclerosis (MS) is an autoimmune disease, having not fully understood aetiology, and both genetic and environmental factors contribute to the pathogenesis of the disease. The cholinergic system has been indicated as a mediator of neuro-immune interactions, as well as an internal regulator of immune responses. The aim of the present research was to assess the associations between BChE and AChE genetic variations and serum cholinergic and inflammatory profiles in 102 Relapsing Remitting-MS patients and 117 healthy controls. An increased frequency of the BChE K-allele in MS patients as compared to controls was found. In addition, data showed that patients had higher BChE enzymatic activity, which is increased by the presence of the polymorphic allele and reduced amounts of circulating ACh. AChE polymorphism was significantly associated to reduced activity in both patients and controls. We propose that serum BChE and AChE activity may be used as a secondary markers to assess the role of non-neuronal cholinergic system in regulating peripheral inflammation via ACh regulation. This pilot study shed light on the role of the non-neuronal cholinergic system in immune cells to better understand MS pathogenesis. The cross-talk between the periphery and the CNS could have a new undescribed crucial role for MS, regarded as a systemic disease.

## Introduction

Multiple Sclerosis (MS) is an autoimmune disease, and even if its aetiology is not yet fully understood, both genetic and environmental factors may contribute to the pathogenesis of the disease^1–3^. MS is considered a chronic autoimmune disease resulting in inflammation and demyelination of the central nervous system (CNS). Its inflammatory phase is characterized by the breaking down of immunological self-tolerance, by episodes of neurological disturbance (relapses) with complete or incomplete remissions, eventually leading to permanent impairment.

The cholinergic neurotransmission is involved in the regulation of the immune response during inflammation. Albeit other autoimmune or neurodegenerative processes could show a similar peripheral immune modification, and peripheral blood markers may not be specific for MS^4^, the blood remains the only readily available biological sample in patients and may help to identify new possible factors involved in the MS pathogenesis^5^. In addition, a crucial role for the cross-talk between the immune and nervous systems has been described. The cholinergic system has been suggested as a mediator of neuro-immune interactions, as well as an internal regulator of immune responses^6,7^.

Previous studies have demonstrated that acetylcholine (ACh) can modify immune responses. Thus, stimulation of the α−7 nAChR (nicotinic cholinergic receptor) by neuronal or non-neuronal ACh, inhibits the release of pro-inflammatory mediators from immune cells by a cholinergic pathway called “anti-inflammatory non-neuronal cholinergic pathway”^8,9^. A comparable regulatory pathway has been described for microglia^10^.

Many studies have indicated that, besides neuronal ACh, non-neuronal cell types such as lymphocytes, macrophages, dendritic cells, adipocytes, keratinocytes, endothelial cells, and epithelial cells, can produce and release ACh and express the ACh-hydrolysing enzymes acetyl-cholinesterase (AChE) and butyrylcholinesterase (BChE), the choline acetyltransferase (ChAT), and acetylcholine receptors of muscarinic and nicotinic type (AChRs)^11–13^. AChE and BChE share approximately 54% amino acid sequence identity. AChE rapidly hydrolyses ACh into acetic acid and choline at extremely fast turnover rates^14^. Alternative functions for AChE have been demonstrated in neuronal and hematopoietic cells such as cell adhesion and proliferation of different types of neurons or regulation of proliferation and apoptosis of multipotent stem cells^15–17^. On the other hand, most tissues and body fluids contain another cholinesterase (ChE) named BChE. Despite the lower catalytic efficiency of BChE than AChE in hydrolysing ACh, BChE contributes to ACh homeostasis as judged by its role in AChE-null mice^18^. The catalytic action of AChE and BChE ensures rapid withdrawal of ACh, which, otherwise, may lead to cholinergic over-activation. Butyrylcholinesterase (BChE) in normal brain and in Alzheimer’s disease (AD) regulates cognitive and behavioural functions^19^. However, the ability of BChE to hydrolyse ACh links this enzyme also to cholinergic neurotransmission and immune modulation. Changes in BChE activity in MS white matter lesions have been described^20^. Myelin, which is composed of a lipid bilayer membrane wraping the axons, is critical for neural signalling and transmission. BChE may be involved in MS in demyelination, due to its “lipolytic” activity, and in neuro-inflammation through acetylcholine hydrolysis. An increase in BChE activity results in reduced acetylcholine levels and absent cholinergic anti-inflammatory responses, that may amplify systemic inflammation^21^.

However, it is difficult to conceive how hyperactivation of the two enzymes with high intrinsic ACh-hydrolysing capacity may have a meaningful pathophysiological impact on ACh levels. In order to balance the actions of highly abundant and efficient cholinesterases, the ACh-synthesizing machinery is however present in peripheral blood cells, whose function is to uphold steady-state equilibrium of ACh levels.

The genetic variations in BChE and AChE genes have been investigated in a number of low-grade systemic inflammation pathologies and in relation to the onset of Alzheimer’s disease^22–26^. In particular, two specific polymorphisms within these genes have been studied, namely rs1803274 for BChE and rs2571598 for AChE. These single nucleotide substitutions have been reported to reduce the enzymes activity of about 30–50% at least for BChE^17,27^. The BuChE rs1803274 is characterized by a G/A substitution inducing the Ala/Thr change at the codon 539. This variation produces the so called K-allele which causes the reduction of 30%-60% of the ACh hydrolysing activity^28^ and 30% of capacity of hydrolysing butyrylthiocholine^29^. The lowered hydrolytic activity of BChE K-allele predicts that BChE K-carriers would potentially sustain improved cholinergic activity. The presence of the K variant of BChE is controversially considered a risk factor for Alzheimer disease (AD). BChE K-carriers are refractory to cholinesterase inhibitor therapy, the current leading treatment for AD^30^.

The AChE rs2571598 is an intronic point mutation characterized by a C/T substitution which affects enzyme activity^31^. Discordant results have been reported about the association between AChE rs2571598 genotype and response of AD patients to AChE inhibitors treatment^31,32^.

We previously reported that serum BChE, AChE and ACh levels differs between MS and healty subjects^33^. The aim of the present research has been to analyse the associations between BChE and AChE genetic variations and altered serum cholinergic and inflammatory profiles. These data could be useful to shed in light the role of cholinergic system in modulating MS inflammation.

## Results

### AChE rs2571598 and BChE rs1803274 genotype and allele frequencies distribution in RR-MS patients and HD

Table 1 compares the genotype and allele frequencies of the AChE rs2571598 in RR-MS patients versus HD controls. Genotype frequencies is according to the Hardy-Weinberg equilibrium, and no statistical significant differences were found in either genotype or allele frequencies distributions.

**Table 1:** AChE rs2571598 genotype and Allele Frequencies in RR-MS patients and HD subjects.

SNP	Genotypes	RR-MS		HD		OR (95% CI)
		n	%	n	%	
AChE rs2571598		102		117		1.73 (1.02–3.00) *p* < *0.05*
	No Carrier	33	32.3	53	45.3	
	Carrier	69	67.7	64	54.7	
	C/C	33	32.3	53	45.3	*p* = *0.068*
	C/T	54	52.9	44	37.6	
	T/T	15	14.8	20	17.1	
Allele frequencies	C	120	58.8	150	64.1	*p* = *0.301*
	T	84	41.2	84	35.9	
HWE (*p-*value)		χ^2^ = 0.49; *p* = 0.5*		χ^2^ = 2.55; p = 0.1*		

However, carrying C/T or T/T genotypes were more frequent in RR-MS patients than in HD controls (67.7% *vs* 54.7%; p < 0.05). The crude OR was 1.73 (95% CI: 1.02–3.00).

Similarly, when comparing the genotype and allele frequencies of the BChE rs1803274 (the so called K-variant) in RR-MS patients versus HD controls, the genotype frequencies is according to the Hardy-Weinberg equilibrium, and no statistical significant differences were found in either genotype or allele frequencies distributions. Carrying G/A or A/A genotypes were more frequent in RR-MS patients than in HD control (49.0% vs 35.9%; p < 0.05). The crude OR was 1.72 (95% CI: 1.01–2.95), (Table 2).

**Table 2:** BChE rs1803274 genotype and Allele Frequencies in RR-MS patients and HD subjects.

SNP	Genotypes	RR-MS		HD		OR (95% CI)
		n	%	n	%	
BChE rs1803274		102		117		1.72 (1.01–2.95) *p* < *0.05*
	No Carrier	52	51.0	75	64.1	
	Carrier	50	49.0	42	35.9	
	G/G	52	51.0	75	64.1	*p* = *0.142*
	G/A	42	41.2	36	30.8	
	A/A	8	7.8	6	5.1	
Allele frequencies	G	146	71.6	186	79.5	*p* = *0.069*
	A	58	28.4	48	20.5	
HWE (*p-*value)		χ^2^ = 0.02 p = 0.9*		χ^2^ = 0.2 p = 0.6*		

### Peripheral cholinergic profiles in BChE K-carrier and no-carrier RR-MS patients and HD subjects

Accordingly with our previous data^34^, BChE activity in serum was different between RR-MS patients and HD subjects, being higher in patients than in control (2510.2 ± 227.4 *vs* 2234.5 ± 192.9; p < 0.001).

**Figure 1:**
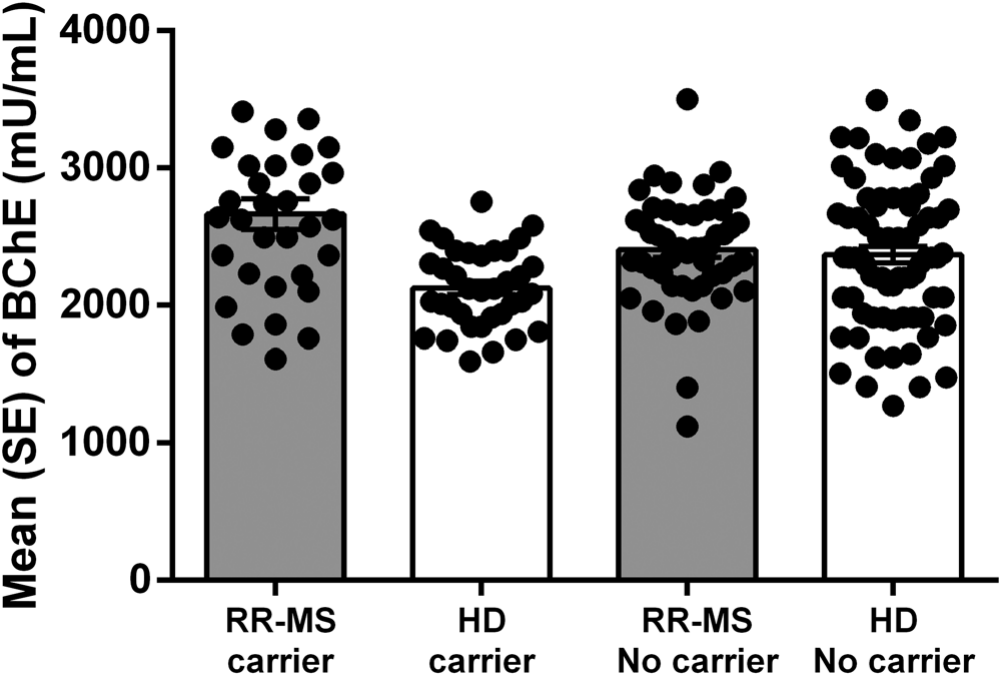
Levels of BChE enzymatic activity in RR-MS patients and HD stratified for presence of BChE K-polymorphism (carrier) and no-carrier for both polymorphism. (Mean ± Standard error; SE).

In K-carriers HD groups we observed a reduction of serum BChE activity, while in K-carriers RR-MS patients an increase of serum BChE activity was detected (Fig. 1). Table 3 shows that in HD, BChE activity was about 10% lower in K-carriers than in no-carriers. In addition, in the HD group, K-carriers had higher levels of ACh (about + 25%), and reduced AChE levels that may be responsible for higher ACh levels observed.

**Table 3:** Levels of cholinergic markers in BChE K-carrier and no-carrier RR-MS patients and HD subjects.

BChE rs1803274	RR-MS		HD		ANOVA p-value		
	Carrier	No Carrier	Carrier	No Carrier	RR-MS vs HD	Carrier vs No carrier	Interaction
BChE (mU/mL)	2757.3 ± 262.6	2418.6 ± 184.1	2121.3 ± 162.2	2348.1 ± 290.7	***0.035***	*0.365*	*0.089*
AChE (mU/mL)	1066.5 ± 843.8	858.2 ± 189.1	420.6 ± 41.6	567.2 ± 145.7	***0.012***	*0.871*	*0.571*
ACh (pmol/mL)	67.7 ± 56.7	98.1 ± 60.1	681.7 ± 418.8	514.0 ± 144.4	<***0.001***	*0.623*	*0.397*

Data are expressed as mean and standard deviation.

In HD the ratio BChE/ACh was respectively 3.1 in K-carriers, and 4.9 in non-carriers. In these subjects, the ratio AChE/ACh was lower, while the ratio BChE/AChE was not significantly different, when compared to non-carrier subjects. Thus, our observation suggest that in HD, the BChE-K allele might have an impact on the AChE activity and ACh levels.

In RR-MS patients BChE activity was about 14% higher in K-carriers than in non-carriers, while AChE activity is higher in K-carriers than in non-carriers. The K-carrier RR-MS patients had lower levels of ACh and the ratio BChE/ACh was 32.5 and 23.7 in K-carrier and non-carrier patients, respectively.

The ratio BChE/ACh may be indicative of the extra-synaptic ACh equilibrium status, since the higher BChE/ACh ratio observed in K-carriers compared to non-carrier RR-MS patients plausibly will lead to less degradative activity of BChE, which is however higher compared to HD K-carrier subjects (Table 4).

**Table 4:** Ratio between cholinergic enzymes activity and ACh levels BChE K-carrier and no-carrier RR-MS patients and HD subjects.

BChE rs1803274	RR-MS		HD	
	Carrier	No Carrier	Carrier	No Carrier
AChE/ACh	15.8 ± 8.2	7.8 ± 3.0	0.7 ± 2.7	1.2 ± 0.8
BChE/ACh	32.5 ± 16.2	23.7 ± 10.9	3.1 ± 1.5	4.9 ± 2.9
BChE/AChE	2.7 ± 0.9	3.5 ± 0.3	5.0 ± 0.2	4.2 ± 0.3
AChE + BChE/ACh	43.7 ± 27.1	24.2 ± 15.7	3.9 ± 2.7	5.8 ± 2.7

Data are expressed as mean and standard error.

### BChE genotype and cytokine levels

Since BChE K-variant is associated with reduced ACh-hydrolysing capacity of the enzyme, and considering the increasing evidence of the immune-regulatory role of ACh, we investigated if genetic heterogeneity in BChE may affect pro-inflammatory cytokines. Particularly, we investigated whether in RR-MS patients and HD subjects, the K allele was related to serum levels of pro-inflammatory cytokines TNFα, IL-17, IL-18 and IL-12/p40.

In HD K-carriers, in accord with lower BChE-hydrolysing capacity and higher ACh levels, we observed lower TNFα, IL-17, IL-18, and IL-12p40 levels when compared to non-carriers HD (Table 5). Thus in HD, BChE genotype exerts regulatory effects on pro-inflammatory cytokines, possibly via regulation of extracellular ACh levels. This finding lends to further support the immune-regulatory function of ACh, that may act as possible negative regulator of inflammation caused by the aberrant T-cell activation.

**Table 5:** Levels of cytokines in serum in BChE K-carrier and no-carrier RR-MS patients and HD subjects.

BChE rs1803274	RR-MS		HD		ANOVA p-value		
	Carrier	No Carrier	Carrier	No Carrier	RR-MS vs HD	Carrier vs no carrier	Interaction
TNFα (pg/mL)	19.4 ± 2.1	16.9 ± 5.6	2.4 ± 0.9	4.8 ± 3.1	***<0.001***	*0.065*	*0.564*
IL-17 (pg/mL)	41.8 ± 13.9	40.4 ± 22.0	10.4 ± 5.2	16.8 ± 3.8	***0.027***	*0.467*	*0.768*
IL-18 (pg/mL)	357.0 ± 63.5	286.7 ± 41.8	168.2 ± 17.1	205.1 ± 22.0	***<0.001***	*0.787*	*0.642*
IL12/p40 (pg/mL)	329.3 ± 41.2	274.4 ± 15.9	76.7 ± 43.2	109.6 ± 36.9	***<0.001***	*0.678*	*0.498*

Data are expressed as mean and standard deviation.

In RR-MS K-carrier patients we observed not significantly differences in inflammatory cytokine levels, probably dependent on the lower ACh levels, as a result of higher levels of both ChE enzymes.

Thus, while BChE K-genotype in HD donors causes the reduction of BChE hydrolysing activity with expected increase of ACh levels and reduced inflammatory environment in the serum, in RR-MS K-carriers the increase of BChE and ACh was associated to an increase of inflammatory cytokines levels.

### Peripheral cholinergic profiles in AChE rs2571598 genotype carrier and non-carrier RR-MS patients and HD subjects

**Figure 2:**
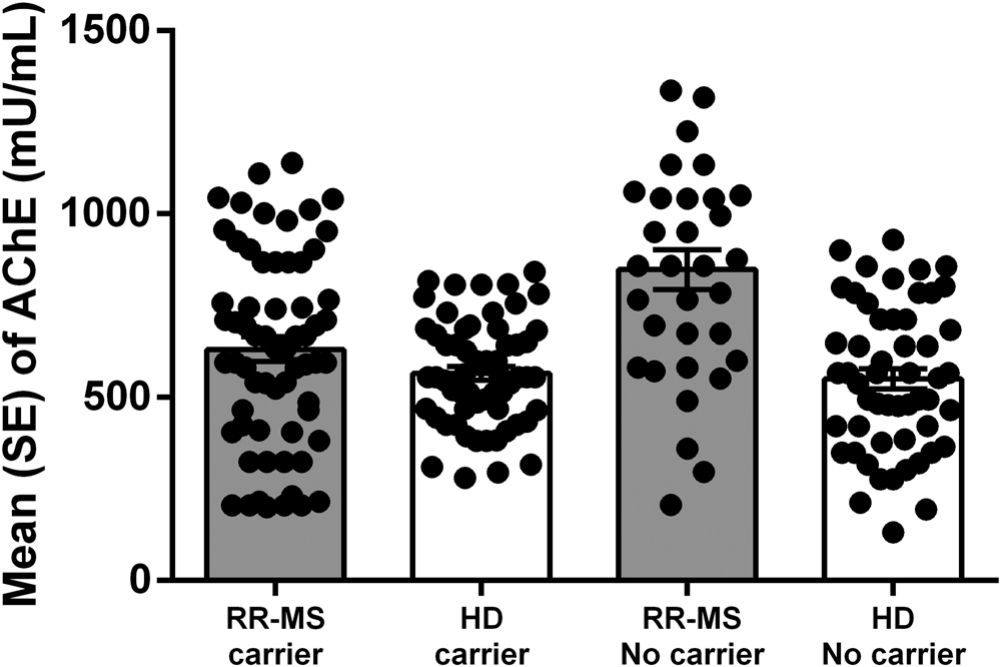
Levels of AChE enzymatic activity in in RR-MS patients and HD stratified for presence of AChE rs2571598 (carrier) and no-carrier for both polymorphism. (Mean ± Standard error; SE).

AChE activity in serum is different between RR-SM patients and HD, showing higher activity in patients (793.5 ± 223.5 *vs* 561.3 ± 100.2; p < 0.001). In both RR-MS patient and HD groups we observed a reduction of AChE activity in serum of AChE rs2571598 genotype carriers (Fig. 2).

In HD carriers of the AChE rs2571598 genotype, ACh levels were 40% higher than in non-carriers. In RR-MS carriers of the AChE rs2571598 genotype, AChE activity was about 30% lower, while ACh levels were significantly higher, than in non-carriers (p = 0.047). The lower levels of ACh, in non-carrier RR-MS patients, probably are modulated by higher AChE and BChE hydrolysing activity (Table 6).

**Table 6:** Levels of cholinergic markers in AChE rs2571598 genotype carrier and no-carrier RR-MS patients and HD subjects.

AChE rs2571598	RR-MS		HD		ANOVA p-value		
	Carrier	No Carrier	Carrier	No Carrier	RR-MS vs HD	Carrier vs no carrier	Interaction
BChE (mU/mL)	2325.9 ± 998.1	2418.6 ± 184.1	2271.7 ± 252.9	2348.1 ± 290.7	*0.227*	*0.654*	*0.943*
AChE (mU/mL)	596.8 ± 543.9	858.2 ± 189.1	556.0 ± 87.2	567.2 ± 145.7	***0.015***	*0.763*	*0.781*
ACh (pmole/mL)	237.1 ± 354.2	98.1 ± 60.1	701.2 ± 345.7	514.0 ± 144.4	***0.004***	***0.047***	*0.652*

Data are expressed as mean and standard deviation.

In HD rs2571598 carriers the ratio AChE/ACh is 1.1 and 1.5 in carriers and non-carriers HD, respectively. In RR-MS patients the AChE/ACh ratio is 3.1 and 7.7 in carriers and non-carriers, respectively (Table 7).

**Table 7:** Ratio between cholinergic enzymes activity and ACh levels in AChE rs2571598 genotype RR-MS patients and HD subjects.

AChE rs2571598	RR-MS		HD	
	Carrier	No Carrier	Carrier	No Carrier
AChE/ACh	3.1 ± 3.7	7.7 ± 2.4	1.1 ± 1.7	1.5 ± 0.8
BChE/ACh	9.9 ± 8.7	25.2 ± 14.1	3.5 ± 2.2	4.5 ± 3.8
BChE/AChE	3.8 ± 0.5	3.1 ± 0.8	4.1 ± 0.2	4.2 ± 0.1
AChE + BChE/ACh	13.1 ± 8.7	32.8 ± 12.7	4.8 ± 0.7	5.5 ± 3.7

Data are expressed as mean and standard error.

### AChE genotype and cytokine levels

In HD rs2571598 carriers, in accord with higher ACh levels, we observed lower TNFα, IL-17, IL-18 and IL-12/p40 levels compared to non-carriers. In addition, RR-MS rs2571598 carriers show lower levels of inflammatory cytokine TNFα and IL-17, and no differences for IL-18 and IL-12/p40 levels. Thus, AChE genotype exerts regulatory effects on pro-inflammatory cytokines, possibly via regulation of ACh levels in both HD and RR-MS. In RR-MS patients carrying rs2571598 both AChE and BChE hydrolysing activities were reduced, parallel to higher ACh levels. In HD rs2571598 carriers, a not significantly reduction of BChE and AChE activity was responsible of a significant increase of ACh levels that may drive the observed reduction of inflammatory cytokines (Table 8).

**Table 8:** Cytokines levels in AChE rs2571598 genotype carrier and no-carrier RR-MS patients and HD subjects.

AChE rs2571598	RR-MS		HD		ANOVA p-value		
	Carrier	No Carrier	Carrier	No Carrier	RR-MS vs HD	Carrier vs no carrier	Interaction
TNFα (pg/mL)	14.3 ± 3.6	16.9 ± 5.6	1.6 ± 1.2	4.8 ± 3.1	***<0.001***	***0.051***	*0.617*
IL-17 (pg/mL)	37.2 ± 18.2	40.4 ± 22.0	9.7 ± 4.8	16.8 ± 3.8	***0.009***	***0.047***	*0.987*
IL-18 (pg/mL)	283.6 ± 52.0	286.7 ± 41.8	168.9 ± 12.9	205.1 ± 22.0	***<0.001***	*0.465*	*0.632*
IL-12/p40(pg/mL)	276.4 ± 33.2	274.4 ± 15.9	80.4 ± 47.6	109.6 ± 36.9	***<0.001***	*0.871*	*0.596*

Data are expressed as mean and standard deviation.

## Discussion

Increasing evidences suggested a role for the cholinergic system in the regulation of the inflammatory pathway. One of the consequences of changes in the ACh equilibrium concerns the immune-regulatory function of cholinergic signalling through the action of ACh on cells involved in native and adoptive immune responses^33–35^. Neuroinflammation in MS brain, initially described as an accumulation of leukocytes, is based upon and is regulated by bidirectional communication pathways involving cytokines that connect the CNS and immune system. Several studies have reported high levels of inflammatory cytokines in MS and have hypothesized the influences of circulating cholinesterases and ACh levels in the serum^34,36,37^. Thus, we have investigated, for the first time, the relationships between the over-production of circulating cholinesterases, the genetic variations of BChE and AChE genes and the levels of circulating cytokines in MS patients.

Our results showed that RR-MS patients have higher serum levels of BChE and AChE compared to HD subjects, and this could explain the reduced amount of circulating ACh observed in patients. We investigated if lower ACh concentration observed in RR-MS compared to HD may be related to the ability to regulate the extracellular ACh, when needed, or to the variation of AChE or BChE activity.

Data showed that AChE rs2571598 and BChE rs1803274 genotype were more frequent in RR-MS patients than in HD control. Results highlighted that in RR-MS patients carrying the K-allele, higher BChE levels were observed, albeit it is in contrast with the reported role of this SNP in BChE gene, and that this over-load of circulating BChE remove ACh, but not sufficiently to reduce efficiently levels of all examined cytokines. Probably, other mechanisms, such as increased transduction of BChE, microRNAs or other genetic factors acting in conjunction with BChE-K, could be involved in the balance of the BChE. The reduced peripheral cholinergic activity, detected in K-carrier HD subjects, may be responsible for the higher ACh levels and for reduction of pro-inflammatory cytokines analysed. The differential levels of ACh detected in K-carriers subjects seem to reflect a physiological adaptation to the ACh-hydrolysing status. Thus, as a relative index for the extracellular ACh equilibrium state, we calculated the ratio of AChE or BChE activity to ACh in the serum of MS K-carrier or no-carrier patients, compared to age-matched controls. An higher BChE + AChE/ACh ratio should indicate a high ACh-hydrolysing status and/or a low ACh-synthesizing status. We found that this ratio is higher in the BChE K-carriers RR-MS group than in non-carriers RR-MS group. In the same group we observed higher levels of the inflammatory cytokines, related to reduced ACh levels. In HD group expressing BChE K-variant, the BChE + AChE/ACh ratio is lower than in non-carriers HD group. When comparing the BChE + AChE/ACh ratio in both RR-MS and HD BChE K-carriers, the ratio resulted higher in patients versus control, in accord with higher inflammatory cytokines levels. This observation strengthen the role of ACh in the control of inflammation^21,36,38^.

In addition our data showed that the BChE K-allele is associated with an increase of circulating AChE in RR-MS, confirming a close relation between the expression of BChE and that of AChE which has long been known^39^. HD show, as expected, increased levels of ACh when the serum levels of BChE and AChE are lower.

Albeit AChE rs2571598 genotype results at least associated with reduced serum AChE levels, more evident in RR-MS patients. The single nucleotide substitution may alter hydrolizing properties, possible for misfolded conformation and formation of a unstable conformation^23^. We found that in both RR-MS patients and HD groups, ACh levels were higher in subjects carrying the polymorphic allele, while no significantly differences in BChE levels were observed. This suggests that AChE rs2571598 genotype may be involved in the lower levels of cytokines detected in serum of AChE rs2571598 carrier subjects, accordingly to lower Ach degradation. This could indicate that AChE genotype may affects the production of inflammatory mediators via regulation of the extracellular levels of ACh. Taken together these data demonstrate that in RR-MS patients the pro-inflammatory immune responses through hydrolysis of acetylcholine could be related to alterations in ChE activity, depending on specific genetic endophenpotypes.

In conclusion, we confirmed that total plasma BChE and AChE activity are higher in RR-MS patients than in healthy controls, an increased frequency of the AChE rs2571598 and BChE rs1803274 in MS patients as compared to controls was found, the plasma ChE activity is associated with ACh levels and with inflammatory status.

Cholinergic system modifications, observed in the periphery could reflect what happens in the CNS, being relevant for immune activation/inflammation, underlining that MS may be considered a systemic disease, where a cross-talk between the periphery and the CNS could have an undescribed crucial role. Furthermore, we propose that serum BChE and AChE activity may be used as a secondary markers to assess the role of non-neuronal cholinergic system in regulating peripheral inflammation via ACh regulation. However, studies on larger populations are needed to confirm the role of non-neuronal cholinergic system in immune cells to better understand MS aetiology and progression and to develop new disease-modifying therapies for MS, with ChE and ACh as targets.

## Methods

### Subjects

Relapsing Remitting-MS (RR-MS) patients were enrolled and followed at Department of Neuroscience of S. Camillo-Forlanini Hospital (Rome, Italy). The diagnosis of RR-MS was confirmed according to revised Mc Donald Diagnostic Criteria^40^ and RR-MS course was established by clinical parameters^41^ in agreement with recent classification^42^. Enrolled patients haven’t been exposed to prior immunomodulatory therapies, such as interferon beta preparations, glatiramer acetate and monthly pulses of intravenously-administered solumedrol; however, they were off these therapies for a minimum of 6 months before the blood collection.

Healthy donors (HD) were enrolled from the Transfusion Blood Bank Services of Chieti (Italy) and were frequency matched for age and gender. Mean age, mean disease duration and EDSS distribution are shown in Table 9. All subjects had not suffered, in the previous month, from inflammatory diseases that might be associated with modulation of cytokines, and had not received corticosteroids or immunosuppressive drugs. All RR-MS patients and HD signed an informed consent. The study was approved by Ethical Committee of San Camillo-Formanini Hospital (prot. n 1457/2016).

**Table 9:** Characteristics of RR-MS patients and HD subjects.

Variable	RR-MS (n = 102)	HD (n = 117)	*p-value*
Age (yr), *mean* ± *SD*	43.0 ± 8.7	47.8 ± 11.2	*0.070*
Gender, *n(%)*			*0.930*
Female	71 (69.7)	80 (68.5)	
Male	31 (30.3)	37 (31.4)	
EDSS, *n(%)*			—
<3	85 (83.2)		
≥3	17 (16.8)		
BOC, *n(%)*			—
Positive	78 (76.8)		
Negative	24 (23.2)		
Duration of disease (yr), *mean* ± *SD*	9.7 ± 6.8		—

### Determination of ACh levels, AChE and BChE Activity

ACh was measured by commercial fluorimetric kit (Abcam, Cambridge, UK), using the Glomax Multi Detection System (Promega, Mi, Italy) at λ Ex/Em 535/587 nm.

Cholinesterases activity were measured in sera of RR-MS patients and HD by Ellman assay^43^, using 1 mM final concentration of acetyl-thiocholine iodide as substrate. In order to evaluate the contribution of AChE and BChE to the total cholinesterase activity, 1.4 × 10^−5^ M BW284c51 or 1.4 × 10^−5^ M lysivane were respectively added as appropriate inhibitors, in the reaction mixture containing 0.33 mM DTNB (di-nitro-thiocyanobenzene) in 0.1 M phosphate buffer, pH 7. Enzyme activity was expressed as mU; 1 mU corresponding to 1 nmole of substrate hydrolysed/min at 30 °C.

### Genotyping

Genomic DNA was extracted from whole blood using the NucleoSpin Tissue kit (Macherey-Nagel, Bethlehem, PA, USA). Genotyping was performed with being blind to case control status for the specific polymorphisms reported in Table 10. The amplification reaction conditions were as follows: denaturation at 94 °C for 10 min followed by 30 cycles at 94 °C for 30 sec, 30 sec at the annealing temperature optimum for each pair of primers (Table 10), 72 °C for 30 sec, with a final elongation step at 72 °C for 10 min.

**Table 10:** Sequences of the primers for BChE rs1803274 and AChE rs2571598.

Polymorphism	Primer Forward	Primer Reverse	Annealing Temperature
BChE rs1803274 (G > A)	ATTAGAGACCCACACAACTT	ATATTTTACAGGAAATATTGATGAA	55
AChE rs2571598 (C > T)	AGAGTCGGGGTCTTGTTATGT	AAAGTGAGGAGGAGACGAGG	60

The specificity of the PCR amplified products was evaluated by 2% agarose gel electrophoresis. The gene polymorphisms were observed by direct sequencing on ABI 3130xl genetic analyzer (Applied Biosystems), on both strands.

Hardy-Weinberg equilibrium (HWE) deviations in the genotype frequency distributions were calculated using the χ square analysis. Hardy-Weinberg-equilibrium-calculator software was used to check for Haplotype association analysis (http://www.had2know.com/academics/hardy-weinberg-equilibrium-calculator-2-alleles.html).

### Cytokine measurements

ELISA assay was conducted with commercial kits (R&D System) according to the manufacturer’s instructions to quantify human cytokine levels in serum. The plates were read at 450 nm and the absorbance was converted in pg/ml, using calibration curves prepared with cytokine standards. The intra- and inter-assay reproducibility were > 90%.

### Statistical analysis

The quantitative variables were summarized as mean and standard deviation (SD) or median and interquartile range (IQR), according to their distribution. Qualitative variables were summarized as frequency and percentage. A Shapiro-Wilk’s test was performed to evaluate the departures from normality distribution for each variable.

The difference in frequencies between patients and controls were assessed by a chi-square test or Fischer exact test when appropriate.

The association between AChE and BChE polymorphisms with SM was assessed by univariate logistic regression analyses. Logistic regression model was applied to estimate the adjusted Odds Ratio (OR) and relative 95% confidence intervals.

The analyses of relationship between genotypes and enzymes activity was performed excluding patients carrying both polymorphisms. Patients with only one polymorphism (carrier group) or without both variants (no-carrier group) were analysed. Two-way analysis of variance (ANOVA) was performed to test the effect of different AChE and BChE genotypes (carrier or no-carrier), different group (RR-MS or HD) and their interaction on levels of BChE, AChE, ACh and cytokines with group as a fixed effect and genotype as a random effect.

The level of statistical significance was set at p < 0.05. Statistical analysis was performed using IBM^®^ SPSS Statistics v 20.0 software (SPSS Inc, Chicago, Illinois, USA).
